# Lidocaine spray vs mepivacaine local infiltration for suturing 1st/2nd grade perineal lacerations: a randomised controlled non-inferiority trial

**DOI:** 10.1186/s12884-024-06640-7

**Published:** 2024-06-24

**Authors:** Stefano Restaino, Matilde Degano, Elisa Rizzante, Ginevra Battello, Federico Paparcura, Anna Biasioli, Martina Arcieri, Gabriele Filip, Luigi Vetrugno, Teresa Dogareschi, Tiziana Bove, Marco Petrillo, Giampiero Capobianco, Giuseppe Vizzielli, Lorenza Driul, LISPRAY Group, Rossana Moroni, Valentina Zanin, Veronica Tius, Lisa Celante, Alessia Sala, Alice Poli, Sara Pregnolato, Giuseppina Seminara, Margherita Cuman, Giulia Pellecchia, Tommaso Occhiali, Cristina Giorgiutti, Stefania Liviero, Ilaria Mazzera, Diana Padovani, Elena De Gennaro

**Affiliations:** 1 Department of Gynecology and Obstetrics, ASUFC University-Hospital of Friuli Centrale, Piazzale Santa Maria Della Misericordia, 15, Udine, UD 33100 Italy; 2 PhD School in Biomedical Sciences, Gender Medicine, Child and Women Health, University of Sassari, Sassari, Sardinia Italy; 3 Department of Medicine (DMED), University of Udine, Via Palladio, 8, Udine, UD 33100 Italy; 4 Department of Anesthesiology, Critical Care Medicine and Emergency, SS. Annunziata Hospital, Via Dei Vestini, Chieti, CH 66100 Italy; 5 Department of Anesthesia and Intensive Care Medicine, ASUFC University-Hospital of Friuli Centrale, Piazzale Santa Maria Della Misericordia, 15, Udine, UD 33100 Italy; 6 Gynecologic and Obstetric Unit, Department of Medical, Surgical and Experimental Sciences, University of Sassari, Piazza Università, 21, Sassari, 07100 Italy; 7 Biostatistician PhD, Independent Research, Piazzale Santa Maria Della Misericordia, 15, Udine, UD 33100 Italy

**Keywords:** Perineal lacerations, Lidocaine spray, Topic anesthetic, Mepivacaine injections, Perineal suturing

## Abstract

**Background:**

Perineal lacerations are a very common complication of post-partum. Usually, the repair of 1st and 2nd-grade lacerations is performed after the administration of local anesthesia. Despite the great relevance of the problem, there are only a few studies about the best choice of local anesthetic to use during suturing. We performed a randomised controlled trial to evaluate the efficacy and safety of the use of a local anesthetic spray during the suturing of perineal lacerations in the post-partum.

**Methods:**

We compared the spray with the standard technique, which involves the infiltration of lacerated tissues, using the NRS scale. 136 eligible women who had given birth at University Hospital of Udine were enrolled and randomly assigned to receive nebulization of Lidocaine hydrochloride 10% spray (experimental group) or subcutaneous/submucosal infiltration of mepivacaine hydrochloride (control group) during suturing of perineal laceration.

**Results:**

The lacerations included 84 1st-grade perineal traumas (61.7%) and 52 2nd-grade perineal traumas (38.2%). All the procedures were successfully completed without severe complications or serious adverse reactions. There were no statistically significant differences between the two groups in terms of blood losses or total procedure time. Moreover, there were no statistically significant differences in terms of NRS to none of the intervals considered. Regarding the application of the spray in the B group, in 36 cases (52.9%) it was necessary to improve the number of puffs previously supposed to be sufficient (5 puffs). Just in 3 cases, an additional injection was necessary (4.4%).

**Conclusions:**

Our study demonstrates that lidocaine spray alone can be used as a first line of local anesthetic during the closure of I-II-grade perineal lacerations, as it has comparable efficacy to mepivacaine infiltration.

**Trial registration:**

The trial was recorded on https://clinicaltrials.gov. Identification number: NCT05201313. First registration date: 21/01/2022. Unique Protocol ID: 0042698/P/GEN/ARCS.

## Background

The trauma most frequently causing lacerations of the perineum is vaginal birth: about 85% of women can suffer post-partum perineal trauma [1–4]. For lacerations of the perineal body and anal sphincter, Sultan et al. in 1999 proposed a grading classification, which is still used today [5, 6]. They divided perineal lacerations into 4 grades, according to the tissue layers involved. First-grade lacerations involve the perineal skin and second-grade ones also involve the perineal muscles. In third- and fourth-grade lacerations, the anal sphincters and rectal mucosa are also affected, respectively.

Usually, the repair of 1st and 2nd-grade lacerations is performed after the administration of local anesthesia. Despite the great relevance of the problem, there is not much evidence in the scientific literature regarding which is the best technique to use [7]. The infiltration of local anesthetic in the perineum is the most common analgesic technique used and the one considered to be the most effective [8]. This procedure allows a significant reduction of pain during suturing, but otherwise, it involves puncturing torn tissues, with further pain for the woman. Considering the high percentage of perineal 1st and 2nd-grade lacerations, it is evident how the use of another route of anesthetic administration could be of great help to further reduce the discomfort of the patient. In particular, the use of lidocaine spray administered through nebulization on the wound could guarantee great clinical efficacy in pain control, pain-free administration, greater ease of use by operators, and the absence of edema in the suture site [9, 10]. Based on our experience, lidocaine spray is frequently used in many delivery rooms as a standalone local anesthetic during perineal lacerations suturing or to enhance the effectiveness of anesthetic injections on the skin. This use, however, is an off-label use [11]. To the best of our knowledge, in fact, lidocaine spray should be used only at the level of the oral mucosa or of the skin, at least within the European Community. Other formulations of topical non-injectable lidocaine such as EMLA cream have been studied with promising results in the setting of postpartum lacerations [8]. The lidocaine spray form has been previously analyzed in the gynecological field for example in Intra-Uterine-Devices (IUD) insertion [12], but to our knowledge no studies exist so far that analyzed the efficacy of lidocaine spray in suturing low grade perineal tears. The rationale of this study is precisely to evaluate the anesthetic efficacy and safety of the nebulization of lidocaine in patients subjected to suturing of postpartum perineal lacerations, by comparing it to the standard technique that involves infiltration of tissues.

## Methods

The study was conducted as a monocentric, prospective, randomised, and controlled trial with two arms. It was an open-label non-inferiority study. In fact, due to the different methods of administration used, it was not possible to guarantee either the blindness of the obstetrician-gynecological team or that of the patients. Only the investigators analyzing the data were blinded to the two groups. The recruitment took place from January to July 2022. Written and verbal information about the study was given to all potentially eligible women during the last antenatal visit and written informed consent was requested from and signed by all participants. Patients were then enrolled in the delivery room or labor room of the Obstetric and Gynecological Clinic of the Santa Maria Della Misericordia University Hospital in Udine, where the inclusion/exclusion conditions were verified. 68 patients were enrolled for each arm of the study for a total of 136 women.

The inclusion criteria included: 1st or 2nd-grade postpartum perineal laceration with the need for suturing; gestational age > 37 weeks, age over 18 years, vertex presentation at the delivery, sufficient ability to understand the language to obtain informed consent, having consented to participate in the study. The exclusion criteria were: having received epidural anesthesia 2 h before the delivery, operative vaginal birth, psychiatric pathology, twin birth, adverse reactions to any local anesthetic in the past, and cardiovascular and/or liver disease during pregnancy including pre-eclampsia. Indeed, after an inner discussion with our dedicated team of anesthesiologists and reviewing the literature [13, 14], we agreed not to exclude all patients who received epidural anesthesia during labor, but only those who still experienced its effects at the time of delivery and suturing. Our anesthesiology team uses 0.1% ropivacaine and 0.25 µg/ml sufentanil boluses as the standard epidural analgesic therapy for labor. The duration of the analgesic effect of these boluses clearly depends on many factors, but on average it lasts about one hour. A two-hour timeframe was found to be sufficiently safe to exclude the interference of epidural analgesia at the time of suturing. Furthermore, before administering the local anesthetic in either form, the NRS score was recorded.

In our clinic, the standard local anesthetic used for perineal infiltration is 20 mg/ml mepivacaine hydrochloride. Therefore, we wanted to compare the use of lidocaine spray with the local anesthetic commonly used in the delivery room. Both lidocaine and mepivacaine are medium-potency local anesthetics of the aminoamide class. Both have a fast onset and provide prolonged analgesia. Additionally, they have comparable toxicity concentrations [10, 15, 16]. So, eligible women were randomly assigned to receive, after wound disinfection and cleansing of bleeding with mild compressive hemostasis: 1) subcutaneous/submucosal infiltration (depending on the type of perineal laceration) of a maximum of 10 ml of 2% mepivacaine hydrochloride 20 mg/ml (control group, A) vs 2) nebulization with 5 puffs of 10 mL Lidocaine hydrochloride 10% spray at 4–5 cm from the mucosa (experimental group, B). In this second case, the device consists of a glass bottle with a dosing pump and a plastic nebulizer dispenser. A single press on the spray head, while holding the device vertically, automatically releases a pre-set dose of lidocaine. The administration of the anesthetic and the suturing of the lacerations were performed by the gynecologist or by the gynecology resident who was working at that time in the delivery room (LISPRAY group), as is routinely done in our Clinic. As there are no previous published studies on the use of nebulized lidocaine for suturing perineal lacerations, the administered number of puffs was chosen according to what is indicated on the lidocaine hydrochloride technical data sheet and in previous studies in the gynecological field, but with other indications [17, 18]. The suture was performed only after the anesthetic took effect, with additional anesthesia administered if the pain exceeded the tolerability threshold. The achievement of adequate anesthesia and the patient’s tolerance were estimated by the same operator who performed the suture, evaluating the patient’s pain through the Numeric Pain Rating Scale (NRS). The NRS is one of the most used and valid methods in the clinical and research fields for the assessment of pain intensity. It consists of a numerical scale from 0 to 10, where 0 corresponds to “no pain” and 10 to “the worst imaginable pain”. Patients were asked to choose the single number that best represents the intensity of their pain [19]. The suture was performed only when the patient reported a score on the scale < 4. The NRS score was based on provoked pain at the beginning of the suturing by pinching tissue with forceps. Starting from the initial 5 predetermined puffs, if the NRS was greater than 4, an additional puff was administered, and the NRS was reassessed until the desired threshold was reached. Once the desired analgesia was achieved, patients were asked to alert the operator if their perception of pain on the NRS scale increased.

The randomisation list was generated with the use of a personal computer and stored on the computer of the delivery room. The patients were enrolled consecutively following the order generated by the list itself. Allocation used a block-randomised computer-generated list. By filling in specific pre-set cards, the data were collected, including the patient’s personal information, data regarding pregnancy, BMI, evidence regarding the type of current laceration and any previous perineal lacerations, the need for additional anesthetic to achieve the absence of pain, the delivery data and the onset of intra- and early post-operative complications. The pain perceived by the patients was then assessed according to the NRS during suturing and at 2, 4, 12, and 24 h after delivery. In our Clinic, pain therapy in the postpartum period usually involves analgesics on demand, upon the patient’s request (1 g of Paracetamol or 600 mg of Ibuprofen). In cases where the timing of the NRS assessment and analgesic administration coincided, the evaluation of pain intensity always occurred before administration. A telephonic follow-up was therefore carried out 30 days after the birth for the registration of the subjective patient satisfaction and of any late postoperative complication [20]. An interview with fixed items regarding the patient’s health status and satisfaction, where 1 corresponded to the lowest satisfaction and 10 to the highest, was asked to the patient. Additionally, questions were asked about any complications, including events such as wound dehiscence, persistent pain or paresthesia, wound infection, hematomas, and any other issues reported by the patient, including also hospital readmissions within 30 days. The primary endpoint of the study was to compare the level of perceived pain during and after the procedure between the two study groups, assessing pain perception at different time points with the NRS scale. Secondary endpoints were the need for additional pain treatment during the suturing and the satisfaction of the patient in the relief of perineal pain, assessed through the NRS and through the final telephonic interview in a 30-days follow-up.

The statistical analysis was conducted starting from a hypothesis of the non-inferiority of the nebulized treatment with respect to subcutaneous infiltration, assuming a clinically relevant mean difference of 1 point or more on the pain rating scale [8, 21, 22] (primary outcome of the study) respect to the experimental group (nebulization). Thus, the sample size was calculated based on objective success rates of 90%. Sample sizes of 68 in Group A and 68 in Group B achieve 90% power to detect a non-inferiority margin difference between the group proportions of -0,1500. The reference group proportion is 0,5000. The treatment group proportion is assumed to be 0,3500 under the null hypothesis of inferiority. The power was computed for the case when the actual treatment group proportion is 0,6000. The significance level of the test is 0,050. The rate of drop out is expected to sum up to 20%. 136 women had to be enrolled for the study. Continuous variables were reported as mean and standard deviations (SD) and categorical variables as numbers and percentages. The two groups were compared concerning their main characteristics to evaluate the efficacy of randomisation. The differences between the two groups for the study outcomes were evaluated with ANOVA or with the Mann–Whitney non-parametric test, depending on the distribution of the data, for continuous variables and with the chi-square test or with the exact test of Fisher, if appropriate, for categorical variables. All the analyses were conducted according to the intention to treat principle. The T-test was used for hypothesis testing. Differences with *p* < 0.05 will be considered significant. The authors will share the data of the study upon request of the Editors.

The trial was conducted after approval from the Regional Ethics Committee (CEUR-2021-Sper-136). Written informed consent was requested for all the recruited women. The trial was recorded on https://clinicaltrials.gov. Identification number: NCT05201313. First registration date: 21/01/2022. Unique Protocol ID: 0042698/P/GEN/ARCS. Study start: 20/01/2022.

## Results

**Fig. 1:**
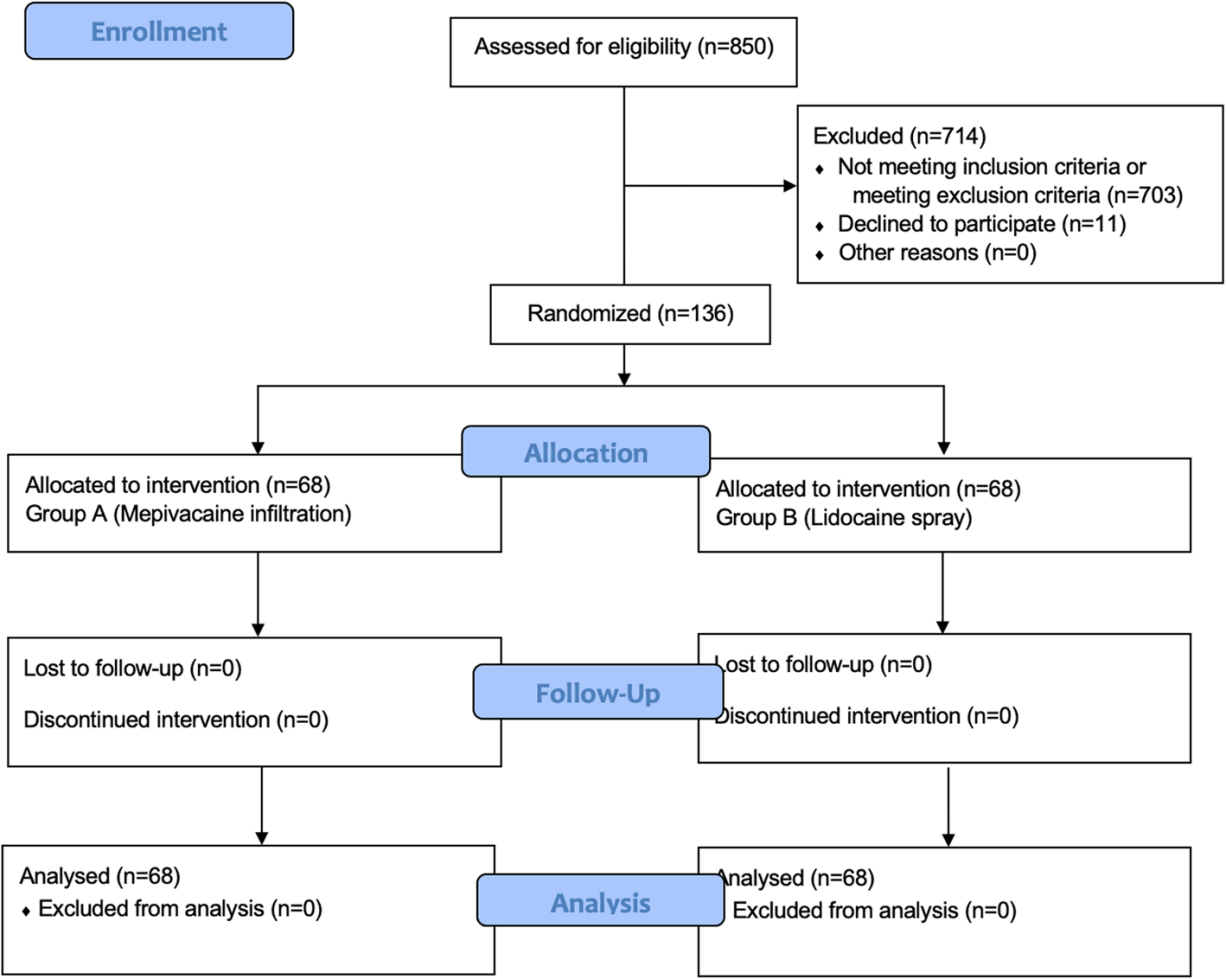
Flowchart of the participants (CONSORT 2012 Statement)

At the time of initial enrollment, 850 women consented to participate in the study and were initially deemed eligible. Among these, at the time of labor/delivery, 136 women were definitively enrolled. There was a dropout of 714 patients who did not meet the inclusion/exclusion criteria: 163 underwent operative vaginal delivery or cesarean section, 67 were excluded due to receiving an episiotomy, 99 because they delivered without perineal tears, 6 due to having a third or fourth-degree laceration, 18 for preeclampsia or other cardiovascular/hepatic conditions, 5 for preterm delivery (< 37 weeks of gestation), 2 patients were < 18 years old, 6 were twins pregnancies, 325 because they had received an epidural anesthesia bolus less than two hours before delivery and 11 declined to participate. 12 patients withdrew their previously given consent at the time of delivery. During the study period, a total of 136 women who met the inclusion criteria and agreed to participate were enrolled. Of these, 68 patients were randomly assigned to receive subcutaneous/submucosal infiltration of mepivacaine (group A) and 68 patients had Lidocaine spray (group B) (Fig. 1). The two groups were similar in baseline characteristics. The median age was 32.2 years in group A and 33.2 years in group B (*p* = 0.87) and the median BMI was respectively 24.9 and 26.4 kg/m^2^ (*p* = 0.93). The lacerations included 84 1st-grade perineal traumas (61.7%) and 51 2nd-grade perineal traumas (37.5%) (Table 1). All the procedures were successfully completed without severe complications or serious adverse reactions. There are no statistically significant differences between the two groups in terms of blood losses (EBL) or total procedure time (OT) (Table 2). There were only 9 mild intraoperative complications. Of these, 6 were in the A group and 3 in the B group. All these complications were postpartum hemorrhages, therefore not related to the method of anesthesia. Only in one case, there was a post-operative complication, and it was a wound hematoma, which resolved spontaneously. It occurred in group B. Furthermore, in the 30-day telephonic follow-up, no patient declared the onset of complications, and all were confirmed to be satisfied with the procedure, with a good general perception of the childbirth experience. Pain scores during and after perineal suturing are shown in Table 2: there is no statistically significant difference in terms of NRS to none of the intervals considered (0, 2, 4, 12, and 24 h). Regarding the application of the spray in the B group (Table 2), in 36 cases (52.9%) it was necessary to improve the number of puffs previously supposed to be sufficient (5 puffs). In particular, 5 patients received only 1 additional puff, 8 patients received 2 additional puffs, 4 patients received 3 additional puffs, and 19 patients received 5 additional puffs. In any case, within a maximum dose of 10 puffs the desired result was achieved. Just in 3 cases, a single additional injection was necessary due to an unsatisfactory anesthetic effect (4.4%). No patient required additional analgesia during suturing: once the NRS < 4 was achieved, it was maintained throughout the suturing time.

**Table 1:** Baseline study population characteristics

Characteristics	N	% or range
All cases	136	
Nulliparas	60	44.1%
Pluriparas	76	55.9%
I° perineal trauma	84	61.7%
II° perineal trauma	51	37.5%
N. of intra-operative complications^a^, ^15^ (%)	9	6.6%
N. of post-operative complications^b^, ^15^ (%)	1	0.73%
Type of anesthesia		
Infiltration	68	50.0%
Spray	68	50.0%
5 puff	29	42.6%
> 5 puff	36	52.9%
Additional injection	3	4.4%

^a^Any adverse or undesirable event occurring during the suture, such as excessive bleeding, injury, or complications caused by the suture itself, or immediate adverse reactions, etc.

^b^Complications occurred after the end of the procedure, found during hospitalization or in the 30-day follow-up (surgical site infections, hematomas, problems with healing, delayed adverse reactions, etc.)

**Table 2:** Comparison of the characteristics and results between the two groups^a^

Characteristics and results	Group A (Mepivacaine infiltration)	Group B (Lidocaine spray)	*p*-value
N. of patients	68	68	/
Mean age (years)	32.2 (22–41)	33.2 (27–42)	0.87
Mean Body Mass Index BMI (Kg/m2)	24.9 (19–42)	26.4 (19–41)	0.93
Mean OT^b^ (range); minutes	13.5 (5–50)	10.9 (2–45)	0.80
Mean EBL^c^ (range); ml	38.9 (0–500)	48.3 (0 -600)	0.94
N. Nulliparas	30 (44.1%)	30 (44.1%)	1.00
N. Pluriparas	38 (55.8%)	38 (55.8%)	1.00
I° perineal trauma	36 (52.9%)	48 (70.5%)	0.052
II° perineal trauma	32 (47.0%)	20 (29.4%)	0.052
Patients who received an epidural bolus more than 2 h before suturing	15	18	0.63
N. of intra-operative complications ^15^ (%)	6 (8.8%)	3 (4.4%)	0.31
N. of post-operative complications^d^^, 15^ (%)	0 (0%)	1 (1.4%)	0.31
Median NRS^e^ score (range)			
Before the anesthetic’s administration	8.5 (5–9)	8.7 (5–10)	0.52
During suturing (t0)	1.5 (0–3)	1.5 (0–3)	0.57
2 h	1.5 (0–9)	1.3 (0–8)	0.93
4 h	1.5 (0–7)	1.3 (0–6)	0.93
12 h	2.1 (0–6)	1.5 (0–7)	0.82
24 h	1.4 (0–7)	0.9 (0–6)	0.83
Perceived satisfaction after 30 days (0–10)	7 (5–8)	7.5 (4–8)	0.06

^a^The differences between the two groups for the study outcomes were evaluated with ANOVA or with the Mann–Whitney non-parametric test, depending on the distribution of the data, for continuous variables and with the chi-square test or with the exact test of Fisher, if appropriate, for categorical variables. All the analyses were conducted according to the intention to treat principle. The T-test was used for hypothesis testing. Differences with *p* < 0.05 will be considered significant

^b^Operative Time

^c^Estimated Blood Loss

^d^Complications occurred after the end of the procedure, found during hospitalization or in the 30-day follow-up (surgical site infections, hematomas, problems with healing, delayed adverse reactions, etc.)

^e^Numeric Rating Scale

## Discussion

Local infiltration of mepivacaine has proven to be an effective method of anesthesia in post-partum tears, however producing great discomfort in the patient [8]. Therefore, this study’s findings confirm the hypothesis of non-inferiority of lidocaine spray compared to mepivacaine infiltration in suturing postpartum 1st and 2nd-grade perineal lacerations. We demonstrated that the anesthetic efficacy of lidocaine spray is comparable to the one of mepivacaine infiltration in this kind of procedure, with a great reduction of patient discomfort. Moreover, lidocaine spray was found to be extremely easy to handle and safe.

Although local anesthetic injections are a common form of topical anesthetic in suturing postpartum lacerations, the other forms of topical anesthetics (sprays, creams, unguents) are becoming increasingly popular in minor surgery just thanks to their ease of use [8]. Other advantages described in the literature in favor of the applications of these local anesthetics are their localized action, which allows excluding adverse effects due to systemic absorption, painless application, and the absence of edema at the application site, which therefore avoids the distortion of suture margins in the repair of lacerations. Furthermore, compared to the infiltration of local anesthetic, there is a lower risk of bleeding [8, 9]. The efficacy of non-injective local anesthetics for minor surgical procedures has already been demonstrated in various fields, both in the obstetric-gynecological and other disciplines [8, 23–28]. In particular, as regards the gynecological procedures, the studies have focused on the effects of various topical anesthetics in the puerperium, of EMLA during suturing of perineal lacerations, of lidocaine spray as anesthetic therapy in the second stage of labor or other gynecological procedures, such as colposcopy, outpatient hysteroscopy, and endometrial biopsies. However, there are currently no studies evaluating the use of lidocaine spray for the repair of perineal lacerations [17, 18, 29–35]. Therefore, previous studies have evaluated the efficacy of other forms of topical anesthetic (for example creams) compared to anesthetic infiltration, in suturing of perineal lacerations, with promising results, but this is the first study demonstrating the efficacy of lidocaine spray in this field [8].

These results seem to us of great relevance given patients’ remarkable perception of pain during the postpartum suture, which creates great discomfort in women [36]. The prevention of obstetric lacerations through assistance techniques for childbirth and preparation of the perineum is certainly the first way to reduce the patient’s distress [37]. However, once the damage has occurred, good local anesthesia helps reduce the negative perception of the childbirth experience.

In this study, there was a high number of excluded patients, most of them due to labor analgesia, which could affect the patient’s perception of pain if administered shortly before suturing. Others were excluded for postpartum perineal integrity or for having received an episiotomy. The two groups were homogeneous regarding the number of epidural analgesia administrations. We excluded only patients who had received a bolus within two hours of suturing (a time frame deemed sufficient for the dissipation of the effect after internal discussion). In both group A and group B, the last bolus had been administered on average 2.7 (2–3.5) and 2.6 (2–3) hours prior. Confirmation of the lack of influence of epidural analgesia on pain perception is supported by the assessment of the NRS before the administration of the anesthetic (Table 2).

Multicentric studies and comparative studies between different forms of topical anesthetics would be desirable in the future. In particular, it would be useful to compare a local anesthetic in the form of a cream, that has already been shown to be effective for suturing perineal lacerations, and lidocaine spray. It would also be desirable to expand the study, also including a comparison of the costs of these products. Furthermore, the studies could also be extended to episiotomies. Moreover, we found that in more than half of the patients allocated to the experimental group, the number of puffs initially considered to be sufficient based on the considered criteria, was actually not. This suggests the need for further studies to determine the exact appropriate dosage. Since there are currently no studies regarding the use of lidocaine spray for suturing perineal lacerations, as for the maximum dosage, there are no precise indications (as also for the number of puffs to administer). According to the product datasheet, no more than 3 puffs per quadrant of oral mucosa should be exceeded (12 puffs). However, no toxic effects have been demonstrated in rats with spray administration, providing us with additional safety data. The research field in this area therefore remains open. The only nearly statistically significant difference between the two groups regarding general characteristics was the number of first and second-degree lacerations (in Group B, there are more first-degree lacerations) (Table 1). This is simply the result of randomization. Perhaps a further suggestion for future studies could be a comparison between first and second-degree lacerations. However, in numerous studies, first and second-degree lacerations are often grouped together [38].

The strength of our study lies in its randomised structure. Its main limitation is the lack of blinding because of the nature of the intervention itself, which is however unavoidable. Furthermore, in our study, we did not include third- and fourth-grade lacerations and the sample size is not large enough to assess the effect of parity and other factors. However, these could still be valuable insights for further studies. It would also be useful to have a placebo-controlled trial in the future, with sham injections and sham application of spray.

## Conclusions

In conclusion, our study demonstrates that lidocaine spray alone can be used as a first line of local anesthetic during the closure of I-II-grade perineal lacerations, as it has comparable efficacy to mepivacaine infiltration. Furthermore, although we do not have strict evidence about this, if an infiltration was still necessary, having previously applied the spray would reasonably appear to help reduce the discomfort of the injection. This is already demonstrated in its use in dentistry on the oral mucosa, as per the product’s package insert. This could be another indication of this local anesthetic also for the genital mucosa.

## Data Availability

The datasets used and/or analyzed during the current study are available from the corresponding author on reasonable request.

## Abbreviations

NRS: Numeric Pain Rating Scale
LISPRAY: LIDOCAINE SPRAY study
